# Neonatal sepsis at Mulago national referral hospital in Uganda: Etiology, antimicrobial resistance, associated factors and case fatality risk

**DOI:** 10.1371/journal.pone.0237085

**Published:** 2020-08-10

**Authors:** Josephine Tumuhamye, Halvor Sommerfelt, Freddie Bwanga, Grace Ndeezi, David Mukunya, Agnes Napyo, Victoria Nankabirwa, James K. Tumwine

**Affiliations:** 1 Centre for Intervention Science for Maternal and Child Health (CISMAC), Centre for International Health, Department of Global Public Health and Primary Care, University of Bergen, Bergen, Norway; 2 Department of Medical Microbiology, Makerere University, Kampala, Uganda; 3 Department of Pediatrics and Child Health, Makerere University, Kampala, Uganda; 4 Department of Public Health, Busitema University, Mbale, Uganda; 5 Department of Epidemiology and Biostatics School of Public Health, Makerere University, Kampala, Uganda; Ross University School of Veterinary Medicine, SAINT KITTS AND NEVIS

## Abstract

**Background:**

Sepsis is the third most common cause of death among neonates, with about 225,000 newborns dying every year globally. Data concerning the microbial etiology of neonatal sepsis and antimicrobial resistance profiles of its causative agents are necessary to inform targeted and effective treatment and prevention strategies.

**Objective:**

To determine the proportion of newborns with symptoms and signs of sepsis who had a positive blood culture, its bacterial etiology, the antimicrobial resistance patterns as well as the factors associated with culture-positivity and case fatality at Mulago national referral hospital in Uganda.

**Methods:**

We conducted a cross-sectional study among 359 neonates with symptoms and signs of sepsis who presented to the pediatric emergency care unit of Mulago national referral hospital from mid-January to end of December 2018. We performed blood culture and antimicrobial susceptibility testing, and conducted polymerase chain reaction to identify methicillin-resistant *Staphylococcus aureus* (MRSA) isolates. We used multivariable logistic regression to estimate the association between potential risk factors and culture-positive neonatal sepsis.

**Findings:**

Of the 359 neonates recruited, 46 (12.8%; 95% CI 9.5%, 16.7%) had a positive blood culture. The predominant isolated bacteria were *Staphylococcus aureus* in 29 (63.0%), *Escherichia coli* in seven (15.2%), and *Klebsiella pneumoniae* in five (10.9%). Of the 46 pathogens, 73.9% were resistant to ampicillin, 23.9% to gentamicin and 8.7% to ceftriaxone. We isolated MRSA from the blood specimens of 19 (5.3%) of the 359 neonates, while 3 (0.8%) grew extended spectrum beta lactamase producers. The case fatality risk among neonates with neonatal sepsis was 9.5% (95% CI: 6.6%, 13.0%). Cesarean section delivery was strongly associated with culture-positive sepsis (adjusted odds ratio 3.45, 95% CI: 1.2, 10.1).

**Conclusion:**

One in eight neonates with clinical signs of sepsis grew a likely causative bacterial pathogen. *S*. *aureus* was the main pathogen isolated and a third of these isolates were MRSA. A significant proportion of the isolated bacterial pathogens were resistant to the first and second line antibiotics used for the treatment of neonatal sepsis. There is need to revisit the current treatment guidelines for neonatal sepsis.

## Introduction

The mortality among children less than 5 years of age has declined considerably over the last two decades, but neonatal mortality remains high, accounting for 2.6 million annual deaths[1]. Uganda has a neonatal mortality of 22.3 deaths per 1,000 live births which is one of the highest in the world[2]. Severe infections are the leading cause and accounts for approximately one-third of newborn deaths in sub-Saharan Africa[3]. Among survivors, neonatal sepsis may be accompanied by long-term complications such as neurodevelopmental impairment[4]. To achieve the third sustainable development goal of reducing neonatal mortality to under 12 deaths per 1,000 live births, a better understanding of the etiology of neonatal sepsis is needed.

Important signs and symptoms of neonatal sepsis include inability to breastfeed, convulsions, fast breathing, severe lower chest wall in-drawing, lethargy, fever and hypothermia[5]. Neonatal sepsis is a clinical syndrome including septicemia and meningitis that is classified according to disease onset[6]. Early-onset sepsis (EOS) is defined as disease among neonates aged 72 hours or less while late-onset sepsis occurs from 4 to 28 days[6]. Early-onset sepsis usually results from an infection acquired *in utero* or during the birth process and group B *Streptococcus*(GBS) is the most common pathogen causing EOS in high income countries whereas *Staphylococcus aureus*, *Klebsiella species* and *Escherichia coli* are the most common causes in low and middle income-countries[7–9]. In low and middle income countries, late-onset sepsis is usually a result of infection from the surrounding environment (hospital or community) and the incriminated pathogens are majorly gram negative bacteria including *E*. *coli*, *Klebsiella pneumoniae*, *Acinetobacter* spp., *Pseudomonas aeruginosa*; as well as *S*. *aureus*[10–12]. However, the pathogen profile differs depending on the region. There is a predominance of gram negative pathogens and low prevalence of GBS in south Asia and sub-Saharan African compared to the high GBS prevalence in high income countries[10, 11, 13]. Surveillance of the etiology of neonatal sepsis and resistance patterns of the causative bacteria is critically important in informing the empirical treatment of neonatal sepsis and in guiding the development of preventive strategies, including the development and deployment of vaccines. This information is particularly important in resource-limited settings where access to blood cultures is limited and if available, often unaffordable.

In Uganda, neonatal sepsis is managed according to the Uganda Clinical Guidelines, which recommend ampicillin and gentamicin as first-line and third-generation cephalosporins as second-line treatment[14]. Although previous studies[15–17] have reported on neonatal sepsis in Uganda, data on the etiology and antimicrobial resistance patterns of the causative bacteria is limited. There is a need to carefully characterize bacterial pathogens from blood cultures of neonates with clinical signs of sepsis because antibiotic susceptibility profiles of the causative organisms change over time[18]. In this study, we aimed to determine the bacterial etiology of culture-positive sepsis and antimicrobial resistance patterns. We also estimated the case fatality risk of neonatal sepsis and factors associated with culture-positive sepsis at Mulago national referral hospital.

## Methods

### Study design and setting

We conducted a hospital-based cross-sectional study at the pediatric emergency care unit of Mulago national referral hospital from January to December 2018. Mulago is the largest tertiary hospital in Uganda, has an average of 100 births per day and its pediatric emergency care unit admits an average of 20 newborns per week. It is the teaching hospital for Makerere University College of Health Sciences, located in Kampala, the capital city of Uganda. The pediatric emergency care unit admits critically ill children for up to 24 hours before they are transferred to the general pediatric wards.

### Participants

We included neonates, i.e. babies less than 29 days of age, who presented with any of the following symptoms and signs during daytime (i.e. 8:00hrs to 17:00hrs) on weekdays: inability to breastfeed, convulsions, fast breathing (more than 60 breaths per minute), severe lower chest in-drawing, lethargy, fever (axillary temperature ≥37.5°C) and low body temperature (axillary temperature<35.5°C) according to the integrated management of childhood illnesses (IMCI) guidelines[19]. We decided to exclude neonates with severe congenital anomalies from the study and those that reported during the night (after 18:00hrs) and (or) during the weekends (Saturday and Sunday).

### Sample size estimation

The sample size was calculated based on the main objective, which was to estimate the proportion of newborns with culture-positive sepsis among children admitted with clinical signs of sepsis. Based on the following assumptions; 95% confidence interval, prevalence of culture proven septicemia 37%[17] and precision 5%, we needed a sample size of 359 newborns with clinical sepsis.

### Study procedure and informed consent

We enrolled neonates who presented with symptoms and signs of sepsis upon admission at the emergency pediatric unit. A trained nurse obtained written informed consent and used validated pretested questionnaires to capture information on demographic and clinical characteristics from mothers or other primary caretakers of the study participants. We collected information on the presence of the following symptoms and signs: inability to feed, convulsions, fast breathing, severe lower chest wall in-drawing, lethargy, fever, hypothermia, umbilical cord stump infection and skin pustules. We also recorded data on the mode of delivery, birth weight, sex of the child, gestational length, antibiotic use before admission, prior hospitalization and its duration. Mothers or caretakers of participants were asked about a history of fever, foul smelling vaginal discharge during their last trimester of pregnancy and premature rupture of membranes. We followed up the participants until they were discharged and collected information which included the length of hospitalization, antibiotics used for treating current illness and immediate clinical outcomes which was defined as either death or recovery.

### Blood specimen collection

Three milliliters of venous blood were drawn aseptically from the participants before the administration of antibiotics. Two milliliters of the blood were inoculated into pediatric blood culture bottles (BD Bactec^TM^ Peds Plus^TM^/F) for culture. The specimens were transported daily to MBN Clinical Laboratories[20].

### Laboratory methods

#### Blood culture

Blood culture bottles were incubated in an automated incubator (BD BACTEC™FX40, USA) at 37°C for 24 hours. Bottles that indicated absence of bacteria in BACTEC were incubated for five more days before considering them negative. When a bottle was flagged positive, blood culture aliquots were sub cultured on blood, chocolate and MacConkey agar (BioLab Budapest, Hungary) and incubated at 35°C to 37°C for up to 96 hours.

#### Identification of bacterial species

Identification of bacterial species was done based on colony morphology, Gram stain appearance and standard biochemical tests[21]. All isolates were stored in 20% glycerol in Brain Heart Infusion broth at -80°C for subsequent molecular identification of methicillin resistant *Staphylococcus aureus* (MRSA).

#### Drug susceptibility testing

Antimicrobial susceptibility testing was performed on the Mueller-Hinton agar using the disk diffusion method as per the Clinical Laboratory Standard Institute (CLSI) guidelines[22]. A lawn culture was made on Muller-Hinton agar plate on which the antibiotic disks were placed and incubated between 35 ^o^C to 37°C for 24 hours. The D-test was performed to detect the presence of erythromycin-induced clindamycin resistance among *S*. *aureus* isolates as previously described[23]. We screened *Enterobacteriaceae* isolates for ESBL resistance using the combination disk method as previously described[24, 25].

Detection of *mecA* gene for the identification of MRSA: Methicillin-resistant *S*. *aureus* (MRSA) was detected using polymerase chain reaction for the *mecA* gene as previously described[26].

#### Quality control

Standard aseptic methods of blood collection and processing were adhered to. *S*. *aureus* ATCC 25923 was used to quality control *S*. *aureus* isolates, *E*. *coli* ATCC 25922 for lactose fermenting bacteria and *P*. *aeruginosa* ATCC 27853 for non-lactose fermenting bacteria. For genotyping, ATCC 25923 (*mecA*-negative) and an in-house *S*. *aureus mecA*-positive strain were used to control for the *mecA* gene.

### Study variables

The main outcome of the study was culture-positive sepsis. A positive blood culture was defined by the isolation of at least one of the following potential pathogens; *S*. *aureus*, *E*. *coli*, *K*. *pneumoniae*, *S*. *pneumoniae*, *N*. *meningitides*, Group B *streptococcus*, *Streptococcus pyogenes*, *C*. *freundii*, *Enterococcus* spp., *Salmonella* spp., *Acinetobacter* spp., *Pseudomonas* spp. and *Enterobacter* spp. A negative blood culture was defined by the absence of bacterial growth or the isolation of possible contaminants such as *Corynebacterium* spp., *Bacillus* spp. and *coagulase-negative Staphylococcus* species. Secondary outcomes included: antibiotic resistance profiles of the isolated pathogens and death of the newborns participating in the study. Other variables used to describe the participants or adjust our estimates were birth weight (dichotomized into low birth weight, i.e. less than 2.5 kg, and normal birth weight, i.e. 2.5 kg or more), age of the newborn, premature rupture of membranes (PROM), umbilical cord stump infection, lethargy, hypothermia, fever, inability to breastfeed, initiation of breastfeeding, previous antibiotic use, re-admission, length of hospitalization, current treatment and presence of resistant pathogens in blood.

### Statistical analysis

We entered data doubly into EPI-Data version 13 and data analysis using STATA 15.0 (StataCorp, College Station, Tx, USA). We summarized categorical variables as proportions and described continuous variables using means and their standard deviations (SD). We performed crude and multivariable logistic regression analysis to assess factors associated with culture-positive sepsis. Crude odds ratios (OR) and 95% confidence intervals (95% CI) were estimated for each exposure variable. Based on previous literature, we decided to include the following variables in our multivariable model; PROM, maternal fever in the last trimester and mode of delivery[27, 28]. Other variables we included were birth weight, umbilical cord infection, prior antibiotic use, foul-smelling vaginal discharge, maternal age and education background. To assess the association between culture-positivity and risk of subsequent death, we estimated the corresponding risk ratio (RR) with a generalized linear model of the binomial family with a log link. Based on existing literature[29, 30], we adjusted the RR for antibiotic treatment before admission, whether the sepsis was of early or late onset, re-admission for the current illness, and mode of delivery.

### Ethical approval and consent to participate

We obtained ethical approval from the Mulago National Referral Hospital Research and Ethics Review Committee (MHREC-1069) and written informed consent from parents/primary caretakers of the neonates enrolled in the study.

## Results

### Description of study participants

None of the neonates considered for inclusion had severe congenital anomalies and, among the 596 babies suspected of having clinical sepsis, we recruited 359 neonates that met our predefined IMCI criteria. Their mean age at admission and recruitment was 8 (SD 7.1) days; about half(53%) of them were admitted within their first, another 27% in their second week of life. Their mean birth weight was 3.1 kg (SD 0.6) and the median duration of hospitalization was 5.4 (IQR 4, 6) days. The mothers of the enrolled neonates had a mean age of 26 years (SD 5.5). Other maternal factors included: fever in the last trimester 105 (29.2%) and foul-smelling vaginal discharge 89 (24.8%) (Table 1).

**Table 1:** Demographic and clinical characteristics of neonates with clinical sepsis at Mulago national referral hospital.

	Culture positive sepsis		
	Yes 46 (%)	No 313 (%)	N = 359 (%)
**Neonatal characteristics**			
age (days)			
0 to 3 (early onset)	15 (32.6)	110 (35.1)	125 (34.8)
4 to 28 (late onset)	31 (67.4)	203 (64.9)	234 (65.2)
**Sex**			
Male	23 (50.0)	178 (57.1)	201 (56.2)
Female	23 (50.0)	134 (42.9)	157 (43.8)
**Birth weight**			
Low	5 (10.9)	43 (13.7)	48 (13.4)
Normal	41 (89.1)	270 (86.3)	311 (86.6)
**Inability to breast feed**			
No	23 (50.0)	163 (52.1)	186 (51.8)
Yes	23 (50.0)	150 (47.9)	173 (48.2)
**Fever**			
No	14 (30.4)	112 (35.8)	126 (35.1)
yes	32 (69.6)	201 (64.2)	233 (64.9)
**Difficulty in breathing**			
No	32 (69.6)	219 (70.0)	251 (70.0)
Yes	14 (30.4)	94 (30.0)	108 (30.1)
**Convulsions**			
No	36 (78.3)	266 (85.0)	302 (84.1)
Yes	10 (21.7)	47 (15.0)	57 (15.9)
**Lethargy**			
No	35 (76.1)	268 (85.6)	303(84.4)
Yes	11 (23.9)	45 (14.4)	56 (15.6)
**Umbilical cord infection**			
No	38 (82.6)	246 (78.6)	284 (79.1)
Yes	8 (17.4)	67 (21.4)	75 (20.9)
**Prior antibiotic use**			
No	34 (73.9)	221 (71.5)	255 (71.8)
Yes	12 (28.5)	88 (28.5)	100 (28.2)
**Maternal characteristics**			
**Age (Years)**			
Less than 20	6 (13.0)	26 (8.3)	32 (8.9)
20–24	17 (37.0)	120 (38.3)	137 (38.2)
25–29	13 (28.7)	90 (28.8)	103 (28.7)
30 or more	10 (21.7)	77 (24.6)	87 (24.2)
**Education**			
Primary	17 (37.0)	105 (33.8)	122 (34.2)
Secondary	23 (50)	170 (54.7)	193 (54.0)
Tertiary	6 (13.0)	36 (11.6)	42 (11.8)
**Mode of delivery**			
Spontaneous vaginal delivery	23 (50)	195 (62.7)	218 (61.1)
Assisted vaginal delivery	17 (37.0)	99 (31.8)	116 (32.5)
Caesarean section	6 (13.0)	17 (5.5)	23 (6.4)
**Marital status**			
Unmarried	6 (13.0)	32 (10.2)	38 (10.6)
Married	40 (87.0)	281 (89.8)	321 (89.4)
**Fever in pregnancy**			
No	38 (82.6)	216 (69.0)	254 (70.7)
Yes	8 (17.4)	97 (31.0)	105 (29.3)
**Foul vaginal discharge**			
No	32 (69.6)	238 (76.0)	270 (75.2)
Yes	14 (30.4)	75 (24.0)	89 (24.8)
**Prolonged rupture of membranes**			
No	34 (73.9)	199 (63.6)	232 (64.9)
Yes	12 (26.1)	114 (36.4)	126 (35.1)

### Bacterial etiology

Of the 359 neonates, 46 (12.8%; 95% CI 9.5%, 16.7%) had a positive blood culture and we did not identify more than one bacterial pathogen in any of them. Of the 46 babies, 15 (32.6%) had early-onset, whereas 31 (67.4%) had late-onset sepsis. Gram-positive bacteria constituted 70% (32/46) of all isolates of which 90.6% (29/32) were *S*. *aureus*. Other gram-positive organisms included *S*. *pneumoniae* 2.2% (1/46) and *Enterococcus* spp. 4.3% (2/46). The remaining 30% (14/46) were gram-negative organisms which included seven *E*. *coli* isolates, five *K*. *pneumoniae* isolates, and one *Neisseria* spp. and *C*. *freundii* isolate (Table 2). We isolated likely contaminants from about 2% (7/359) of the neonates. The likely contaminants included 2 *Bacillus* spp., 4 *Corynebacteria* spp., and one isolate of coagulase-negative *Staphylococcus*.

**Table 2:** Bacterial pathogens isolated from blood of neonates with symptoms and signs sepsis at Mulago national referral hospital.

Bacteria Isolated	Overall (%)	Early onset (0–3 days)	Late onset (4-28days)
*S*. *aureus*	29 (8.1)	13 (7.0)	16 (10.0)
*E*. *coli*	7 (2.0)	5 (2.7)	2 (1.2)
*K*. *pneumoniae*	5 (1.4)	3 (1.6)	2 (1.2)
*Enterococcus* spp.	2 (0.6)	2 (1.1)	0
*Neisseria* spp.	1 (0.3)	0	1 (0.6)
*S*. *pneumoniae*	1 (0.3)	0	1 (0.6)
*C*. *freundii*	1 (0.3)	0	1 (0.6)
**Total**	46	23	23

### Antimicrobial resistance profiles of isolated bacteria

We found that among the 46 neonates with a positive blood culture, resistance to first-line antibiotics was identified in ampicillin 39 (84.8%) and gentamicin in 11 (23.9%), while resistance to second-line 3^rd^ generation cephalosporins was identified in 4 (8.7%). Overall, the proportions of babies with likely causative bacteria that were resistant to first-line and to second-line antibiotics were 13.9% (50/359, 95% CI; 10.5, 17.9) and 1.1% (4/359, 95% CI; 0.3, 2.8) respectively. Two thirds of the babies who had blood cultures with *S*. *aureus* had MRSA, while erythromycin-induced clindamycin-resistant *S*. *aureus* were observed in31%. All the *S*. *aureus* isolates were susceptible to vancomycin. Forty-one percent (19/46; 95% CI 27.0%, 56.8%) of the babies with culture-positive sepsis had MRSA. Overall, the proportion of participants with MRSA was 5.3% (19/359; 95% CI 3.2%, 8.1%).

All *K*. *pneumoniae* and six *E*. *coli* isolates were resistant to ampicillin. Resistance to gentamicin was observed in two (28.6%) of seven *E*. *coli* isolates and in two (40%) of five *K*. *pneumoniae* isolates. Three of the *K*. *pneumoniae* isolates, one *E*. *coli* and one *Neisseria* spp. isolates were resistant to third-generation cephalosporins (ceftriaxone and ceftazidime). All gram-negative bacteria isolated in this study were susceptible to the carbapenem class of antibiotics (Table 3). However, three bacterial pathogens phenotypically exhibited ESBL resistance translating to a proportion of 6.5% (3/46; 95%CI 1.4%, 17.9%) among neonates with positive-culture sepsis. Enterobacteriaceace that exhibited carbapenem resistance included one *E*. *coli* and three *K*. *pneumoniae* isolates. Overall, the proportion of the neonates with MRSA was 5.3% (19/359; 95% CI 3.2%, 8.1%), with ESBL producers 0.8% (3/359; 95% CI 0.02%, 2.4%) and with carbapenem resistance 1.1% (95% CI 0.3%, 2.8%).

**Table 3:** Antimicrobial resistance profile of pathogens isolated from neonates with symptoms and signs of sepsis at Mulago national referral hospital between January and December 2018.

Antibiotics	All participants	Neonates with culture-positive sepsis	*S*. *aureus*	*E*. *coli*	*K*. *pneumoniae*
	N = 359 (%)	n = 46 (%)	n = 29	n = 7	n = 5
Erythromycin	21 (5.8)	21 (45.7)	21(72.4)	NA	NA
Vancomycin	0	0	0	NA	NA
Tetracycline	13 (3.6)	13 (28.3)	13 (44.8)	NA	NA
Penicillin	28 (7.8)	28 (60.9)	28 (96.6)	NA	NA
Gentamicin	11 (3.1)	11 (23.9)	7(24.1)	2 (28.6)	2 (40.0)
Trimethroprim-Sulphamethoxazole	34 (9.5)	34 (73.9)	27 (93.1)	4 (57.1)	3 (60)
Chloramphenicol	8 (2.2)	8 (17.4)	7 (15.2)	0	1 (2.2)
Ampicillin	39 (10.9)	39 (84.8)	28 (96.6)	6 (85.7)	5 (100.0)
Amoxycillin-clavulanic acid	9 (2.5)	9 (19.6)	NA	5 (71.4)	4 (80.0)
Ceftazidime*	4 (1.1)	4 (8.7)	NA	1 (14.3)	3 (60)
Ceftrioxone*	4 (1.1)	4 (8.7)	NA	1 (14.3)	3 (60)
Cefuroxime*	4 (1.1)	4 (8.7)	NA	1 (14.3)	3 (60)
Imipenem*	4 (1.1)	4 (8.7)	NA	1 (14.3)	3 (60)

*The four isolates (resistant to these antibiotics) are the same.

### Factors associated with culture-positive sepsis

The odds of being born by caesarian section among neonates with culture-positive sepsis was three times that among babies born by spontaneous vaginal delivery (AOR 3.45, 95% CI; 1.19,10.05). Subgroup analysis yielded similar findings (Table 4).

**Table 4:** Neonatal and maternal factors potentially associated with culture-positive sepsis among neonates with symptoms and signs sepsis at Mulago national referral hospital.

Neonatal characteristics	Culture-positive sepsis (0–28 days)		Culture-positive sepsis (0–7 days)*		Culture-positive sepsis (0–14 days)*	
	Unadjusted OR (95% CI)	Adjusted OR (95% CI)	Unadjusted OR (95% CI)	Adjusted OR (95% CI)	Unadjusted OR (95% CI)	Adjusted OR (95% CI)
Age (days)						
0 to 3	1	-	-	-	-	-
4 to 28	1.12 (0.58, 2.16)	-	-	-	-	-
Birth weight						
Normal	1	1	1	1	1	1
Low	0.77 (0.29, 2.05)	0.84 (0.30, 2.31)	1.03 (0.29, 3.71)	1.35 (0.34, 5.31)	1.29 (0.43, 3.88)	0.91 (0.29, 2.89)
Umbilical cord infection						
No	1	-	1	1	1	-
Yes	0.77 (0.34, 1.74)	-	0.59 (0.21, 1.63)	0.55 (0.18, 1.66)	0.62 (0.26, 1.47)	-
Prior antibiotic use						
No	1	-	1	-	1	-
Yes	0.79 (0.46, 1.36)	-	0.80 (0.33, 1.94)	-	0.88 (0.55, 1.43)	-
Maternal characteristics						
Age (Years)						
Less than 20	1.63 (0.59, 4.53)	1.43 (0.49, 4.18)	0.69 (0.14, 3.38)	0.59 (0.11, 3.11)	1.33 (0.44, 4.01)	1.17 (0.38, 3.65)
20–24 (Reference group)	1	1	1	1	1	1
25–29	1.02 (0.47, 2.21)	1.0 (0.45, 2.22)	0.71 (0.25, 2.00)	0.67 (0.22, 2.09)	0.80 (0.34, 1.85)	0.74 (0.31, 1.78)
30 or more	0.92 (0.40, 2.11)	0.74 (0.30, 1.79)	0.78 (0.27, 2.22)	0.52 (0.16, 1.66)	0.85 (0.36, 2.04)	0.74 (0.29, 1.88)
Education						
Primary	1.03 (0.38, 2.79)	1.11 (0.39, 3.19)	1.17 (0.29, 4.67)	1.39 (0.31, 6.21)	1.12 (0.38, 3.34)	1.19 (0.38, 3.76)
Secondary	0.86 (0.33, 2.25)	0.89 (0.30, 1.79)	1. 11(0.30, 4.16)	1.14 (0.27, 4.72)	0.99 (0.35, 2.84)	1.00 (0.33, 3.03)
Tertiary	1	1	1	1	1	1
Mode of delivery						
Spontaneous vaginal delivery	1	1	1	1	1	
Assisted vaginal delivery	1.47 (0.75, 2.88)	1.59 (0.79, 3.18)	1.54 (0.64, 3.71)	1.82 (0.70, 4.76)	1.30 (0.63, 2.66)	-
Caesarean section	3.02 (1.10, 8.43)	3.45 (1.19, 10.05)	3.58 (0.84, 15.17)	4.45 (0.92, 21.52)	2.78 (0.81, 9.61)	-
Fever in pregnancy						
No	1	1	1	1	1	1
Yes	0.47 (0.21, 1.04)	0.47 (0.21, 1.07)	0.28 (0.08, 0.96)	0.20 (0.05, 0.76)	0.44 (0.19, 1.04)	0.37 (0.15, 0.91)
Foul vaginal discharge in pregnancy						
No	1	1	1	1	1	1
Yes	1.18 (0.84, 1.66)	1.30 (0.90, 1.88)	1.27 (0.83, 1.95)	1.53 (0.94, 2.48)	1.18 (0.83, 1.69)	1.43 (0.97, 2.12)
PROM						
No	1	1	1	1	1	1
Yes	0.61 (0.31, 1.22)	0.53 (0.25, 1.11)	0.53 (0.21, 1.31)	0.46 (0.17, 1.25)	0.58 (0.27, 1.26)	0.47 (0.21, 1.04)

*Analysis restricted to 0–7 and 0–14 day old babies.

### Immediate clinical outcomes

Of the 359 neonates with symptoms and signs of sepsis, 34 died, translating to a case fatality risk of 9.5% (95% CI; 6.6%, 13.0%). The case fatality risk for culture-positive sepsis was 15.2% (7/46; 95% CI 6.3%, 28.9%) in comparison to 8.6% (27/313; 95% CI 5.8%, 12.3%) among the culture negative participants. After adjusting for previous antibiotic use, age, re-admission and mode of delivery, the odds of death among neonates with culture-positive sepsis were 1.6 times higher than that among those with culture-negative sepsis (adjusted RR 1.60; 95% CI 0.66, 4.0).

Of the 34 neonates that died, 25 (73.5%) were treated with only first-line antibiotics (ampicillin and gentamicin) while 9 (32.4%) were treated with second-line antibiotics (ceftazidime, ceftriaxone and cefixime). Pathogens isolated from newborns that died included three *S*. *aureus* isolates (two of which were MRSA), two *E*. *coli* isolates (one of which was an ESBL producer), one ESBL producing *K*. *pneumoniae* isolate and one *C*. *freundii* isolate. Regarding the length of hospitalization among those who died, 30 neonates were hospitalized for ≤5 days, two neonates for 14 days, one for 16 and one for 18 days.

## Discussion

This study aimed to determine the proportion of neonates admitted with signs and symptoms of clinical sepsis to the pediatric department of the Ugandan national referral hospital in Kampala who had a positive blood culture. It aimed to also describe the etiology of culture-positive sepsis in these newborns and the antimicrobial resistance profiles of isolated bacteria and the factors associated with culture-positive sepsis.

The predominate pathogens isolated from neonates with symptoms and signs of sepsis were *S*. *aureus*, *E*. *coli* and *K*. *pneumoniae*. Our findings were similar to those from previous studies in East Africa[5, 31]. Unlike our study, *Klebsiella* spp. and *K*. *pneumoniae* were the most commonly isolated bacterial species in blood drawn from neonates with clinical sepsis in a hospital-based Zambian study, possibly explained by a nosocomial outbreak[32]. Generally, many African studies report gram-positive bacteria, especially *S*. *aureus*, as the main cause of neonatal sepsis; a situation very different from that in South Asia where gram-negative bacteria, especially *Acinetobacter* spp. and *K*. *pneumoniae* seem to be the main cause[10, 33]. The predominance of *S*. *aureus* among neonates with culture-positive sepsis in our study may indicate vertical transmission, more so because this pathogen was also common among babies with early onset sepsis.

We observed resistance to antibiotics (ampicillin, gentamicin and ceftriaxone) commonly used to treat neonatal sepsis. Ampicillin and gentamicin were the first-line antibiotics used to treat almost all of the neonates in our study. Among the bacterial pathogens isolated from the blood of participants, we observed almost three quarters were resistant to ampicillin and almost one quarter to gentamicin. This is similar to what other studies in Tanzania[31], Ethiopia[34] and Zambia[32] have reported. The resistance patterns observed in our study are not surprising because the pathogens adapt to selection pressure exerted by the misuse of common antibiotics[35]. *K*. *pneumoniae* and *E*. *coli* isolates have intrinsic antimicrobial resistance mechanisms which include chromosomally encoded antibiotic inactivating enzymes or efflux pumps; that enable the many gram-negative bacteria to exhibit such resistance to ampicillin/penicillin antibiotics[36].

Our findings are comparable to those in Kenya[37] and Nigeria[38, 39]. On the contrary, a Ugandan study previously conducted at the Mulago hospital reported resistance among the bacterial pathogens to gentamicin to be lower than that in our study[17]. Since that study was conducted more than 15 years ago from the same hospital as our study, the differences observed between the findings may be explained by a remarkable and worrying acquisition of antimicrobial resistance genes among important invasive bacterial pathogens. Four of 14 (29%) gram-negative pathogens in our study were resistant to third-generation cephalosporins. The proportions of resistance to third-generation cephalosporin in our study were similar to those observed in other African studies[34, 40].

ESBL producers accounted for 6.5% of the isolated pathogens. Other studies in low-income settings reported proportions of ESBL pathogens ranging from 10.5% to 25% among pathogens from neonates with sepsis[41–44]. Although the proportion of ESBL producing bacteria was relatively low in our study, it was imprecisely estimated, and their isolation in the blood of neonates with sepsis is important. This is because ESBL pathogens are associated with long hospitalization stays and death[45, 46]. In-fact, two of the 3 neonates with ESBL in this study died.

Most of the *S*. *aureus* strains isolated in this study were methicillin resistant. The high proportion of MRSA among *S*. *aureus* isolates suggests the possibility of transmission from the colonized maternal genital tract or transmission from the labour ward and neonatal units after unhygienic personal or obstetric practices. Susceptible *S*. *aureus* bacteria adapt to antimicrobial pressure through the acquisition of mobile genetic elements carrying antimicrobial resistance genes from other bacteria encoding modified penicillin binding protein 2a which has very low affinity for beta-lactam drugs[47].

In this study, we found that the odds of developing culture-positive sepsis among neonates born through cesarean section were three times that of those born through vaginal delivery. Although statistically imprecise, our findings are similar to those from other studies[34, 48] that found cesarean-section to be associated with neonatal sepsis. This finding is not surprising, especially in settings with poor infection control practices. In addition, this association may be explained by underlying indications for cesarean-section such as obstructed labour, premature rapture of membranes and prolonged labor. The immature immune system of neonates puts them at high risk of infection, especially those born prematurely or with low birth weight and who are not breastfeeding[49]. In contrast to other studies[50, 51], we did not find strong associations between low birth weight, premature rupture of membranes and culture-positive neonatal sepsis.

### Strengths and limitations

Recruiting neonates from the national referral hospital over a one-year period may be a relatively good representation of neonates with clinical symptoms and signs of sepsis in Kampala and the surrounding areas. However, our study was too small to yield statistically precise estimates for the less common pathogens and for the resistance patterns for the less common ones. Further, it had inadequate power to fully explore the association between the exposures and neonatal sepsis. As most other studies, we will have underestimated the proportion of neonates with neonatal septicemia, both because blood culture in itself has a poor sensitivity[52] and because we did not collect cerebrospinal fluid to diagnose meningitis.

## Conclusion

An eighth of neonates admitted to the national referral hospital with clinical signs of sepsis had a positive blood culture. *S*. *aureus* was the most commonly isolated pathogen, and two-thirds of such isolates were MRSA. Resistance to first-line antibiotics used for the management of sepsis was common. Our neonates with culture-positive sepsis had a high case fatality risk, and there is therefore an urgent need for quick and accurate diagnostic tools for systemic bacterial infections in neonates. Our and others’ findings that caesarian section seems to be associated with culture-positive sepsis indicates that health workers need to be alerted if babies born to mothers with such delivery fall sick.

## Supporting information

S1 Dataset Dataset de-identified. (XLSX)
